# Associations Among Cardiometabolic Risk Factors, Sleep Duration, and Obstructive Sleep Apnea in a Southeastern US Rural Community: Cross-Sectional Analysis From the SLUMBRx-PONS Study

**DOI:** 10.2196/54792

**Published:** 2024-11-08

**Authors:** Adam P Knowlden, Lee J Winchester, Hayley V MacDonald, James D Geyer, John C Higginbotham

**Affiliations:** 1 Department of Health Science The University of Alabama Tuscaloosa, AL United States; 2 Department of Kinesiology The University of Alabama Tuscaloosa, AL United States; 3 Institute for Rural Health Research College of Community Health Sciences The University of Alabama Tuscaloosa, AL United States; 4 Research Institute of Pharmaceutical Sciences The University of Mississippi University, MS United States

**Keywords:** obstructive sleep apnea, obesity, adiposity, cardiometabolic, cardiometabolic disease, risk factors, sleep, sleep duration, sleep apnea, Short Sleep Undermines Cardiometabolic Health-Public Health Observational study, SLUMBRx study

## Abstract

**Background:**

Short sleep and obstructive sleep apnea are underrecognized strains on the public health infrastructure. In the United States, over 35% of adults report short sleep and more than 80% of individuals with obstructive sleep apnea remain undiagnosed. The associations between inadequate sleep and cardiometabolic disease risk factors have garnered increased attention. However, challenges persist in modeling sleep-associated cardiometabolic disease risk factors.

**Objective:**

This study aimed to report early findings from the Short Sleep Undermines Cardiometabolic Health-Public Health Observational study (SLUMBRx-PONS).

**Methods:**

Data for the SLUMBRx-PONS study were collected cross-sectionally and longitudinally from a nonclinical, rural community sample (n=47) in the southeast United States. Measures included 7 consecutive nights of wrist-based actigraphy (eg, mean of 7 consecutive nights of total sleep time [TST_7N_]), 1 night of sleep apnea home testing (eg, apnea-hypopnea index [AHI]), and a cross-sectional clinical sample of anthropometric (eg, BMI), cardiovascular (eg, blood pressure), and blood-based biomarkers (eg, triglycerides and glucose). Correlational analyses and regression models assessed the relationships between the cardiometabolic disease risk factors and the sleep indices (eg, TST_7N_ and AHI). Linear regression models were constructed to examine associations between significant cardiometabolic indices of TST_7N_ (model 1) and AHI (model 2).

**Results:**

Correlational assessment in model 1 identified significant associations between TST_7N_ and AHI (*r*=–0.45, *P*=.004), BMI (*r*=–0.38, *P*=.02), systolic blood pressure (*r*=0.40, *P*=.01), and diastolic blood pressure (*r*=0.32, *P*=.049). Pertaining to model 1, composite measures of AHI, BMI, systolic blood pressure, and diastolic blood pressure accounted for 25.1% of the variance in TST_7N_ (*R*^2^_adjusted_=0.25; *F*_2,38_=7.37; *P*=.002). Correlational analyses in model 2 revealed significant relationships between AHI and TST_7N_ (*r*=–0.45, *P*<.001), BMI (*r*=0.71, *P*<.001), triglycerides (*r*=0.36, *P*=.03), and glucose (*r*=0.34, *P*=.04). Results from model 2 found that TST_7N_, triglycerides, and glucose accounted for 37.6% of the variance in the composite measure of AHI and BMI (*R*^2^_adjusted_=0.38; *F*_3,38_=8.63; *P*<.001).

**Conclusions:**

Results from the SLUMBRx-PONS study highlight the complex interplay between sleep-associated risk factors for cardiometabolic disease. Early findings underscore the need for further investigations incorporating the collection of clinical, epidemiological, and ambulatory measures to inform public health, health promotion, and health education interventions addressing the cardiometabolic consequences of inadequate sleep.

**International Registered Report Identifier (IRRID):**

RR2-10.2196/27139

## Introduction

Insufficient sleep has emerged as a critical public health burden [1,2]. More than 35% of adults in the United States identify as short sleepers, receiving less than 7 hours of sleep per night [3]. Among this sample of short sleepers, more than half of men and over one-third of women self-reported snoring [3], a potential indicator of obstructive sleep apnea (OSA)—a sleep-mediated breathing disorder characterized by 5 or more apnea-hypopnea events per hour [4]. Short sleep and OSA are underrecognized burdens on the public health infrastructure [2,3]. More than 80% of individuals in the United States with OSA remain undiagnosed [5]. Furthermore, in Western cultures, a sufficient sleep duration of 7-9 hours of sleep each night [6] is often perceived as a dispensable luxury, contributing to a significant portion of short sleep being voluntary in nature [7].

A growing body of evidence [8] suggests short sleep and OSA are associated with cardiometabolic disease risk factors including adiposity [9], hypertension [10,11], hypercholesterolemia [12,13], and hyperglycemia [14,15]. Studies have found that short sleep increases sympathetic outflow to the heart, leading to increased blood pressure (BP) [16], while OSA is linked to various cardiovascular events such as secondary and resistant hypertension [17], ischemic heart disease [18], stroke [19], and arrhythmias [11]. Associations between desaturation episodes and hyperlipidemia in OSA-affected patients have also been observed [13], and short sleep is correlated with increased total and low-density lipoprotein cholesterol (LDL-C) levels in nonclinical populations [20]. Chronic intermittent hypoxia, a result of OSA, increases the risk of glucose intolerance [14], and a single night of partial sleep restriction can potentiate insulin resistance, even in apparently healthy adults [7,15].

The relationship between obesity and inadequate sleep [9] has garnered increased attention due to their overlapping prevalence [7], potential causal relationship [21,22], and hypothesized interaction with cardiometabolic etiology [9,23]. Proposed mechanisms for the sleep-obesity association include dysregulation of satiation-signaling hormones leading to overeating [24] and daytime fatigue impeding motivation for physical activity [9].

While progress has been made in identifying sleep-associated risk factors for cardiometabolic disease, challenges persist [25,26]. Epidemiological studies investigating sleep generally rely on retrospective, cross-sectional, self-report, nonvalidated measures [3,27], often yielding mixed results. Experimental studies of sleep, while methodologically rigorous, frequently recruit small sample sizes [28] and terse observation periods [29], limiting their generalizability to naturalistic, free-living conditions [30].

To model the relationship between sleep and cardiometabolic disease risk factors, studies incorporating rigorous measures of sleep collected under naturalistic conditions are required. In line with this objective, we report preliminary findings from the Short Sleep Undermines Cardiometabolic Health-Public Health Observational study (SLUMBRx-PONS). SLUMBRx-PONS was designed to encompass a broad spectrum of clinical, epidemiological, and ambulatory measures, collected both cross-sectionally and longitudinally.

## Methods

### Data Collection

The current study represents the first analysis of data collected as part of SLUMBRx-PONS. The methods for the SLUMBRx-PONS have been detailed elsewhere [31]. Clinical data were collected cross-sectionally at the University of Alabama Exercise Science Laboratory (Tuscaloosa, Alabama). Epidemiological and ambulatory data were collected longitudinally over a 7-night period using an internet-based, web portal (Hypknowledge website). At the conclusion of the 7-night data collection period, participants returned to the study location to submit unused study equipment, obtain copies of their health data in report format, and receive a financial incentive.

### Recruitment and Consent

Participants for the study were recruited by distributing flyers throughout the community and through research study advertising networks established by the University of Alabama Division of Strategic Communication (Tuscaloosa, Alabama). An internet-based screening questionnaire was built using survey software (Qualtrics [Silver Lake]) to determine study eligibility. Study inclusion criteria limited enrollment to respondents who were (1) at least 18 years of age; (2) with a permanent home address that is located within proximity to the study site; (3) who are currently employed; (4) that currently operate a motor vehicle; (5) with reliable access to the internet and to a secure PC, laptop, or tablet; (6) with a valid email address and mobile phone number; and (7) who are committed to completing all study activities. Health-related exclusionary study criteria included (1) pregnancy, (2) classified as underweight (BMI<18.5 kg/m^2^), (3) prescription of any sleep medications, (4) continuous positive airway pressure therapy, (5) diagnosis of heart disease, or (6) diabetes.

### Variables

#### Anthropometric-Based Biomarkers

Participant body weight was measured in a fasted state to 0.05 kg using a calibrated, electronic scale (COSMED). Height was measured to the nearest 1 mm using a stadiometer (SECA) without shoes and with light clothing. Height (in m) and weight (in kg) were used to calculate BMI (kg/m^2^) with reference ranges categorized as (1) underweight, <18.5 kg/m^2^; (2) normal weight, 18.5-24.9 kg/m^2^; (3) overweight, 25.0-29.9 kg/m^2^; and (4) obese, 30.0 kg/m^2^.

#### Cardiovascular-Based Biomarkers

Systolic blood pressure (SBP) and diastolic blood pressure (DBP) were measured in accordance with published guidelines [32]. Briefly, the same trained clinician measured resting BP in a seated position after a 10-minute rest period in a quiet room using an automated device (SphygmoCor XCEL [AtCor Medical Pty Ltd]). BP readings were measured in duplicate, with 2 minutes between readings, on the right arm, with an appropriately sized cuff while the brachial artery was supported at heart level. The average of 2 BP readings was considered and used for analysis if SBP and DBP values agreed within 5 mm Hg; up to 6 additional BP readings were obtained until agreement was achieved (if agreement was not achieved within 6 readings, all values were averaged and used for analysis). Resting BP was classified using the following reference ranges: (1) normal, SBP <120 mm Hg and DBP<80 mm Hg; (2) elevated, SBP 120-129 mm Hg and DBP<80 mm Hg; (3) stage 1 hypertension, SBP 130-139 mm Hg or DBP 80-89 mm Hg; and (4) stage 2 hypertension, SBP≥140 mm Hg or DBP≥90 mm Hg [32].

#### Blood-Based Biomarkers

Blood samples were collected from fasted participants by a trained phlebotomist for measures of cholesterol (lipid panel) and glucose. Samples were taken in a seated position and analyzed immediately (Abbott Cholestech LDX Analyzer). Lipids and glucose were expressed as mg/dL with reference ranges categorized as (1) high-density lipoprotein cholesterol (HDL-C), low <40 mg/dL (men), low <50 mg/dL (women); (2) triglycerides, normal: <150 mg/dL, borderline high: 105-199 mg/dL, high: 200-499 mg/dL; (3) LDL-C, desirable: <100 mg/dL, less than desirable: 100-129 mg/dL, borderline high: 130-159 mg/dL, high: 106-189 mg/dL, very high: ≥190 mg/dL; and (4) fasting glucose, desirable: <100 mg/dL, impaired: 100-125 mg/dL, diabetes mellitus: ≥126 mg/dL [33].

### Sleep Parameters

#### Sleep Duration

Sleep duration was measured over 7 consecutive nights using wrist-based actigraphy (FitBit Inspire 2 [Google]). Each morning, participants synced the wearable device to the standardized study web portal (Hypknowledge website). Sleep duration was operationalized as the mean of 7 consecutive nights of total sleep time in minutes (TST_7N_) and categorized as (1) short sleep duration, TST_7N_<420 minutes and (2) normal sleep duration, TST_7N_≥420 [6,34].

#### Obstructive Sleep Apnea

During their visit to the exercise physiology science laboratory, participants were provided with a home sleep test (HST) recording device. The first 30 participants were fitted with ReactDx HSTs. The AccuSom HST was conducted outside of a clinical center using an AccuSom Type III diagnostic device. This device simultaneously recorded several parameters, including oral and nasal airflow, chest wall motion (measured by impedance), oxygen saturation (using a pulse oximeter), heart rate, and snoring. The apnea-hypopnea index (AHI) values were calculated by dividing the total number of apneas plus hypopneas by the total monitoring time and Respiratory Event Index (REI) values were calculated by dividing the total number of respiratory events by the total monitoring time [35]. The monitoring time was defined as the recorded time minus any excluded bad data or artifacts, in accordance with the American Academy of Sleep Medicine guidelines. As described and in accordance with American Academy of Sleep Medicine guidelines, the calculation of AHI is based on monitoring duration rather than actual sleep time. The second set of participants (n=17) were fitted with the Itamar Medical WatchPAT ONE HST [36]. The WatchPAT ONE HST is worn on the wrist and uses a plethysmography-based, finger-mounted probe that measures the PAT (peripheral arterial tone) signal. The PAT signal reflects pulsatile volume changes in the fingertip arteries, which indicate the relative state of arterial vasomotor activity and indirectly, the level of sympathetic activation. Peripheral arterial vasoconstriction, which corresponds to sympathetic activation, appears as attenuation in the PAT signal amplitude. In addition, snoring, body position, and chest movement signals are recorded by the integrated chest sensor. Following the sleep study, the recordings are automatically downloaded from the web server and analyzed using the proprietary zzzPAT software (Itamar Medical), which calculates respiratory parameters, including the peripheral AHI. The peripheral AHI represents the average number of apneas (complete pauses in breathing) and hypopneas (partial reductions in airflow) per hour of sleep, based on peripheral arterial tone, heart rate, oxygen saturation, and actigraphy, rather than traditional airflow measurements [37].

Participants were instructed on device use and were provided with contact information for device support should any issues arise with their use of the HST. The HST raw data, in its entirety, were reviewed and interpreted by a board-certified sleep specialist and physician (JDG). OSA diagnosis was determined using AHI values with reference ranges categorized as (1) none or minimal, AHI<5; (2) mild, AHI≥5, but <15; (3) moderate, AHI≥15, but <30; and (4) severe, AHI≥30 [38].

### Statistical Analyses

A total of 2 independent researchers (APK and Josh Williams) from the SLUMBRx-PONS laboratory team cross-checked all data for accuracy and completeness. The significance for statistical assessment of all results was set a priori at the .05 level. Pearson correlation coefficients (*r*) were calculated to evaluate the relationships between the anthropometric (BMI), cardiovascular (SBP and DBP), and metabolic (HDL-C, LDL-C, triglycerides, and glucose) indices and the TST_7N_ and AHI variables. Variables with significant correlations were used to construct linear regression models examining associations between the significant cardiometabolic indices and the TST_7N_ (model 1) and AHI (model 2) sleep variables.

Given the use of nonprobability sampling and the smaller sample size analyzed, violations of model-building assumptions were anticipated. Subsequently, diagnostics were conducted to evaluate normality (ie, Shapiro-Wilk test or histogram inspection), linearity (ie, test for linear trend or scatterplot inspection), homoscedasticity (ie, Breusch-Pagan test or P-P plot inspection), and multicollinearity (ie, variance inflation factor or matrix scatterplot inspection). To address violations, inverse transformation of nonnormal variables was applied. As well, multivariate outliers identified using Mahalanobis distance (chi-square *P*=.05) were removed. Finally, bias-corrected and accelerated bootstrapping with 10,000 samples was performed on the linear regression models to construct robust 95% CIs for the regression coefficients [39]. Data analysis was performed using IBM SPSS (version 29.0) and Microsoft Excel (version 2311). Data are presented as mean and SD, unless otherwise noted.

### Ethical Considerations

Enrolled participants were provided copies of the study’s institutional review board–approved informed consent documentation before data collection (IRB 19-04-2288). All deidentified electronic data were stored on the University of Alabama’s HIPAA (Health Insurance Portability and Accountability Act)-compliant cloud service. For the in-laboratory component of the study, participants received no-cost access to the data collected during their laboratory visit. For the in-home component, payment was prorated as follows: (1) 7 nights of 100% complete sleep diaries, US $50; (2) 7 nights of 100% complete activity monitor entries, US $50; (3) 7 (100%) complete survey sets; and (4) valid and complete HST, US $50. All participants who completed 100% of the home-based portion of the study were provided a US $200 financial incentive for their participation.

## Results

### Participants

**Figure 1:**
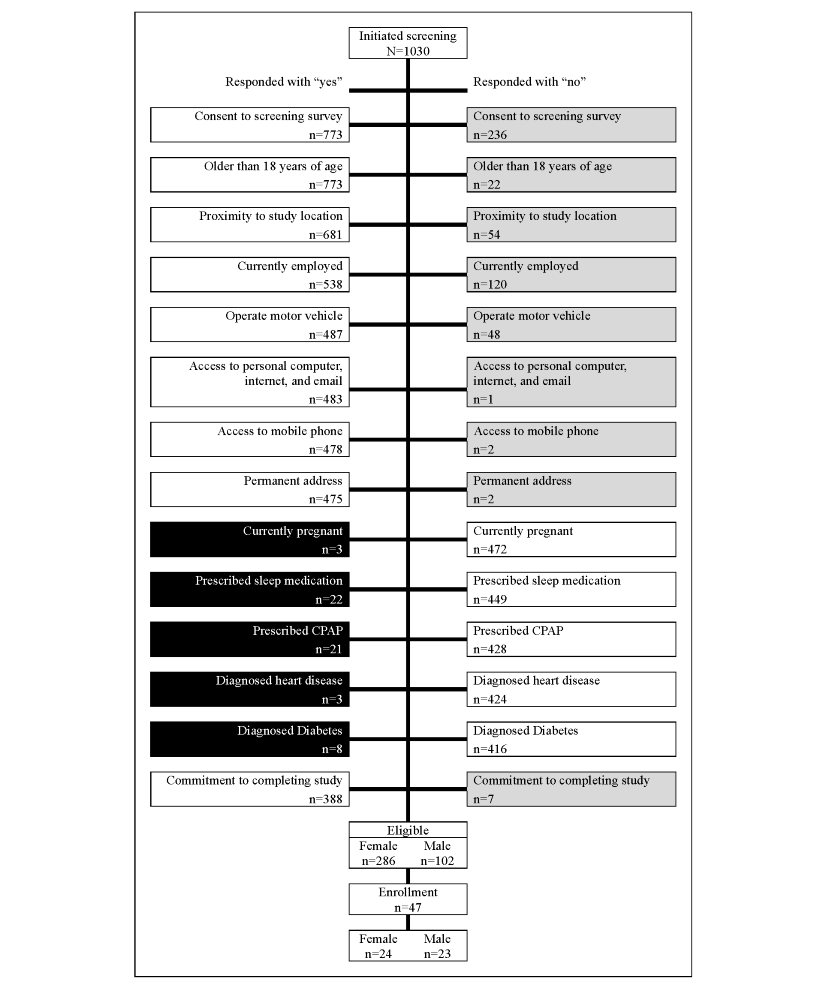
Short Sleep Undermines Cardiometabolic Health-Public Health Observational study onboarding flow chart (n=1030). CPAP: continuous positive airway pressure.

Participant screening was conducted using an internet-based enrollment survey, which included a series of yes or no questions to determine inclusion and exclusion criteria. Figure 1 illustrates the flow of study respondents through the screening process. Unshaded boxes represent participants who met the study inclusion criteria, while gray-shaded boxes indicate respondents excluded due to participation-related factors. Black shaded boxes represent those excluded for health-related reasons. Discrepancies in the frequency totals across screening criteria are due to some respondents discontinuing the survey.

Out of the 1030 respondents who initiated the study enrollment survey, 388 were eligible to participate in SLUMBRx-PONS at the time of this analysis. Among the eligible respondents, more women (n=286) than men (n=102) initiated and completed the intake screening questionnaire. Given the nearly 2:1 ratio of females to males enrolled in the study and the deadlines to process participants, a sex-balanced enrollment approach was used based on the date the participant completed the survey. Under this strategy, for each female enrolled, 1 male was also enrolled, resulting in an equivalent balance of men and women in the study sample.

After sex-matching, enrollment was then prioritized based on BMI-matching. For each female in the participant pool, the first enrolled was classified as normal weight BMI, the second as overweight BMI, and the third as obese BMI. A similar strategy was attempted for each male in the participant pool. However, BMI categories were not equivalent during this initial reporting period, resulting in unbalanced BMI categories during the early reporting period. Of the 388 eligible participants, 47 were enrolled and analyzed for this study. No participants were lost to follow-up, ensuring that all data were used in the analysis.

The enrolled participants were roughly equivalent between female (n=24) and male (n=23), identifying as African American or Black (n=6), Asian (n=8), Caucasian or White (n=31), and multiracial or other (n=2). In addition, 45 participants identified as non-Hispanic. As a group, participants were overweight (based on BMI) with mildly elevated BP and overall normal blood biomarker levels. In terms of sleep parameters, participants displayed symptoms of mild OSA and experienced short sleep duration. Only 4 participants experienced oxygen saturation below 90% (T90%).

On average, both women and men were short sleepers, and nearly half experienced mild (n=8 women and n=9 men), moderate (n=2 women and n=3 men), or severe (n=1 woman and n=1 man) OSA. More women were categorized as normal weight (n=11) and obese (n=8) compared with men (n=8 and n=5, respectively), while a higher proportion of men (n=10) were overweight relative to women (n=5). Sex-based differences in cardiometabolic disease risk factors were not significantly different between men and women. However, there were trends between several, including HDL-C and triglycerides, although the results were not statistically significant (Table 1).

Multivariate outliers were removed (n=8) before constructing the correlational matrix. Model 1 correlational assessment identified significant associations between TST_7N_ and AHI (*r*=–.45, *P*=.004), BMI (*r*=–.38, *P*=.02), SPB (*r*=.40, *P*=.01), and DPB (*r*=.32, *P*=.049). Model 2 correlational analyses found significant relationships between AHI and TST_7N_ (*r*=–.45, *P*<.001*)*, BMI (*r*=.71, *P*<.001), triglycerides (*r*=.36, *P*=.03), and glucose (*r*=.34, *P*=.04). Table 2 displays the correlational coefficient matrices for the SLUMBRx-PONS study participants.

**Table 1:** Descriptive statistics of the cardiometabolic disease risk factors and sleep indices from Short Sleep Undermines Cardiometabolic Health-Public Health Observational study participants (N=47; female n=24 and male n=23).

Variable			Minimum		Maximum		Mean (SD)		M Rank^a^		*U^b^*		*P* value
**BMI (kg/m^2^)**													
	Female	18.82		43.16		28.73 (7.66)		24.96		253.0		.63	
	Male	19.50		41.94		27.00 (5.88)		23.00		—^c^		—	
	Total	18.82		43.16		27.89 (6.83)		—		—		—	
**AHI^d^**													
	Female	0.80		42.00		7.47 (9.32)		21.27		210.5		.16	
	Male	1.20		53.80		9.88 (11.27)		26.85		—		—	
	Total	0.80		53.80		8.65 (10.28)		—		—		—	
**TST_7N_^e^**													
	Female	195.86		552.56		397.17 (80.37)		25.58		238.0		.42	
	Male	324.08		490.50		386.91 (40.82)		22.35		—		—	
	Total	195.86		552.56		392.15 (63.66)		—		—		—	
**SBP^f^ (mm Hg)**													
	Female	106.00		154.70		122.13 (11.95)		25.06		250.5		.59	
	Male	107.30		141.30		119.94 (10.23)		22.89		—		—	
	Total	106.00		154.70		121.06 (11.07)		—		—		—	
**DBP^g^ (mm Hg)**													
	Female	66.30		97.00		78.57 (8.21)		25.58		238.0		.42	
	Male	62.70		99.70		76.97 (8.53)		22.35		—		—	
	Total	62.70		99.70		77.79 (8.31)		—		—		—	
**HDL-C^h^ (mg/dL)**													
	Female	16.00		88.00		52.75 (15.18)		27.31		196.5		.09	
	Male	16.00		91.00		47.22 (15.13)		20.54		—		—	
	Total	16.00		91.00		50.04 (15.25)		—		—		—	
**LDL-C^i^ (mg/dL)**													
	Female	55.00		182.20		109.01 (32.22)		24.44		265.5		.82	
	Male	68.00		200.20		108.13 (33.99)		23.54		—		—	
	Total	55.00		200.20		108.58 (32.74)		—		—		—	
**Triglycerides**													
	Female	45.00		143.00		79.67 (30.88)		20.56		193.5		.08	
	Male	45.00		181.00		102.30 (44.17)		27.59		—		—	
	Total	45.00		181.00		90.74 (39.25)		—		—		—	
**Glucose**													
	Female	69.00		121.00		89.38 (11.96)		22.81		247.5		.54	
	Male	70.00		103.00		89.96 (7.99)		25.24		—		—	
	Total	69.00		121.00		89.66 (10.10)		—		—		—	
**Age (years)**													
	Female	20.00		62.00		34.88 (11.69)		25.21		247.0		.54	
	Male	19.00		61.00		33.39 (12.48)		22.74		—		—	
	Total	19.00		62.00		34.15 (11.97)		—		—		—	

^a^M Rank: Mean Rank.

^b^*U*: Mann-Whitney *U* Test statistic.

^c^Not applicable.

^d^AHI: apnea-hypopnea index.

^e^TST_7N_: mean of 7 consecutive nights of total sleep time.

^f^SBP: systolic blood pressure.

^g^DBP: diastolic blood pressure.

^h^HDL-C: high-density lipoprotein cholesterol.

^i^LDL-C: low-density lipoprotein cholesterol.

**Table 2:** Correlational coefficient matrix of the mean of 7 consecutive nights of total sleep time in minutes and cardiometabolic risk factor indices from the Short Sleep Undermines Cardiometabolic Health-Public Health Observational study participants (n=39).

	TST_7N_^a^	AHI^b^	BMI	SBP^c^	DBP^d^	HDL-C^e^	LDL-C^f^	Triglycerides	Glucose	Age
TST_7N_	—^g^									
AHI	–0.45^h^									
BMI	–0.38^i^	0.71^h^								
SBP	–0.41^h^	0.31	0.20							
DBP	–0.35^i^	0.31	0.07	0.70^h^						
HDL-C	0.22	–0.06	–0.02	0.06	0.05					
LDL-C	–0.17	0.09	0.17	0.08	0.34^i^	–0.05				
Triglycerides	–0.27	0.36^i^	0.29	–0.14	0.14	–0.44^h^	0.42^h^			
Glucose	–0.16	0.41^i^	0.39^i^	0.10	0.11	–0.19	0.13	0.23		
Age	0.15	–0.11	0.04	0.13	–0.14	0.06	0.03	0.002	0.07	

^a^TST_7N_: mean of 7 consecutive nights of total sleep time.

^b^AHI: apnea-hypopnea index.

^c^SBP: systolic blood pressure.

^d^DBP: diastolic blood pressure.

^e^HDL-C: high-density lipoprotein cholesterol.

^f^LDL-C: low-density lipoprotein cholesterol.

^g^Not applicable.

^h^Significance level *P*<.01.

^i^Significance level *P*<.05.

### Main Results

Linear regression models were constructed to explore the relationships between significant cardiometabolic disease indices and the sleep parameters: TST_7N_ (Model 1) and AHI (Model 2). During assumption testing, multicollinearity was detected between AHI and BMI. Due to the strong association between these variables among SLUMBRx-PONS participants (*r*=0.71, *P*<.001) and their historically established linear relationship [40], principal component analysis (PCA) was used to create a new composite variable representing the shared variance between AHI and BMI (PCA_AHI×BMI_) [41]. Similarly, PCA was used to transform SBP and DBP into a single composite measure of BP (PCA_BP_), due to their strong association (*r*=0.70, *P*<.001) and previous applications [42]. Along with standardizing AHI, BMI, SBP, and DBP for PCA, all remaining variables in the regression models including TST_7N_ (*z*_TST7N_), triglycerides (*z*_TRG_), and glucose (*z*_GLU_) were converted to *z* scores to calculate standardized bootstrapped regression coefficients.

Model 1 analysis found that PCA_AHIxBMI_ (β=–.36, bias-corrected and accelerated [BCa] 95% CI –0.58 to –0.17, *P*=.02) and PCA_BP_ (β=–.32, BCa 95% CI –0.55 to –0.08, *P*=.04) accounted for 25.1% of the variance in *z*_TST7N_ (*R*^2^_adjusted_=0.25; *F*_2,38_=7.37, *P*=.002). For every 1-unit increase in *z*_TST7N_, PCA_AHI×BMI_ decreased by 0.36 units. Similarly, for each 1-unit increase in *z*_TST7N_, PCA_BP_ decreased by –0.32 units *z*_TST7N_=[–0.36×PCA_AHIxBMI_]+[–0.32×PCA_BP_]). Analysis of Model 2 found *z*_TST7N_ (β=–.30, BCa 95% CI –0.54 to –0.11, *P*=.02), *z*_TRG_ (β=.32, BCa 95% CI 0.05-0.53, *P*=.02), and *z*_GLU_ (β=.42, BCa 95% CI 0.16-0.66, *P*=.02) accounted for 37.6% of the variance in PCA_AHI×BMI_ (*R*^2^_adjusted_=0.38; *F*_3,38_=8.63, *P*<.001). For each 1-unit increase in PCA_AHI×BMI_, *z*_TST7N_ decreased by –0.30 units. Conversely, for each 1-unit increase in PCA_AHI×BMI_, *z*_TRG_ increased by 0.32 units and *z*_GLU_ increased by 0.42 units (PCA_AHI×BMI_=[–0.30× *z*_TST7N_]+[0.32× *z*_TRG_]+[0.42×*z*_GLU_]). Table 3 summarizes the bootstrapped standardized regression coefficients of the cardiometabolic disease risk factors and sleep indices regression models.

**Table 3:** Bootstrapped standardized regression coefficients for the cardiometabolic disease risk factors and sleep indices regression models from the Short Sleep Undermines Cardiometabolic Health-Public Health Observational study (n=39; unless otherwise noted, bootstrap results are based on 10,000 bootstrap samples).

Variable			B^a^		Bias		SE B		*P* value		BCa^b^ 95% CI
***z*_TST7N_^b,c,d^**											
	PCA_AHIxBMI_^e^	–.36		.000		0.11		0.003		–0.58 to –0.17	
	PCA_BP_^f^	–.32		.001		0.12		0.01		–0.55 to –0.08	
**PCA_AHI×BMI_^e,g,h^**											
	*z*_TST7N_^b^	–.30		–0.02		0.12		0.02		–0.54 to –0.11	
	*z*_TRG_^i^	.32		–0.002		0.13		0.02		0.06 to 0.53	
	*z*_GLU_^j^	.42		–0.003		0.13		0.002		0.16 to 0.66	

^a^B: standardized coefficient.

^b^BCa: bias-corrected and accelerated.

^b^*z*_TST7N_: standardized mean of 7 consecutive nights of total sleep time.

^c^For *z*_TST7N_ model, *R*^2^_adjusted_=0.25; *F*_2,38_=7.37, *P*=.002.

^d^*z*_TST7N_=(–0.36×PCA_AHI×BMI_)+(–0.32×PCA_BP_).

^e^PCA_AHI×BMI_: composite measure of apnea-hypopnea index and BMI derived from principal component analysis.

^f^PCA_BP_: composite measure of systolic blood pressure and diastolic blood pressure derived from principal component analysis.

^g^For PCA_AHI×BMI_ model, *R*^2^_adjusted_=0.38; *F*_3,38_=8.63, *P*<.001.

^h^PCA_AHI×BMI_=(–0.30× *z*_TST7N_)+(0.32× *z*_TRG_)+(0.42×*z*_GLU_).

^i^*z*_TRG_: standardized mean of triglycerides.

^j^*z*_GLU_: standardized mean of glucose.

## Discussion

### Anthropometric-Based Biomarkers

In tandem with the majority of cross-sectional and longitudinal investigations of body composition, sleep duration, and OSA [43], early findings from the SLUMBRx-PONS study identified significant associations among these covariates. Short sleep duration is hypothesized to interact with obesity through a series of causally connected bidirectional pathways [44]. Short sleep can lead to a dysregulation of the hormones that control hunger and satiation, resulting in excessive caloric consumption [45]. In addition, fatigue resulting from short sleep can reduce behavioral intentions for physical activity [46]. In addition to short sleep, OSA is hypothesized to operate as both an independent and comorbid risk factor of obesity. The Wisconsin Sleep Cohort study [47] observed for each 1-SD increase in BMI, the odds of OSA increased 4-fold. Physiologically, excess adipose contributes to OSA through fat deposition in the tissues surrounding the upper airway, resulting in a smaller lumen and increased collapsibility of the upper airway [48]. Risk factors for obesity resulting from short sleep and OSA amalgamate, often concurrently, perpetuating a further increased risk for obesity, which, in turn, potentiates further disordered sleep.

### Cardiovascular-Based Biomarkers

Among the cardiovascular biomarkers, the composite BP measure (PCA_BP_) was significantly associated with TST_7N_. The relationship between TST_7N_ and BP likely involves several physiological mechanisms. Sleep duration–associated risk factors of coronary heart disease include heightened sympathetic overactivity and hypertension [16]. Physiologically, BP operates diurnally and dips between 10% and 20% during sleep [49]. Subsequently, short sleep has been found to acutely increase 24-hour BP. Chronic sleep deprivation has been associated with hemodynamic alterations, posited to induce hypertrophic remodeling of the arterial walls and left ventricle [49], contributing to entrained elevated BP [10].

OSA was not associated with SBP or DBP in the current analysis; however, emerging trends were observed, although not statistically significant. Given a larger sample size, the final study results may yield associations between OSA and measures of BP. Elevated AHI is linked to the development and exacerbation of hypertension through several mechanisms. Recurrent episodes of apnea and hypopnea lead to intermittent hypoxia, which triggers sympathetic nervous system activation and consequent surges in BP [50,51].

This sympathetic overactivity persists during wakefulness, contributing to long-term hypertension [52]. In addition, intermittent hypoxia induces oxidative stress and inflammation, further exacerbating endothelial dysfunction and hypertension [53]. The severity of OSA, as indicated by AHI, appears directly proportional to the risk of developing hypertension [11]. Peppard et al [51] demonstrated a dose-response relationship between AHI and the incidence of hypertension, where individuals with severe OSA (AHI>30) were at a significantly higher risk compared with those without OSA. Furthermore, the Wisconsin Sleep Cohort study [54] found that each SD increase in AHI was associated with a 4-fold increase in the odds of developing hypertension [51].

### Blood-Based Biomarkers

Short sleep has been hypothesized to mediate hypercholesterolemia through appetite equilibrium disruption, daytime fatigue, and sympathetic nervous system activation [16,55]. OSA influences hypercholesterolemia through similar mechanisms as short sleep; however, OSA is posited to increase the risk of dyslipidemia through unique pathways exclusive to its pathogenesis [13]. Metabolically, both short sleep and OSA contribute to glucose-impairing indices including sleep fragmentation [56], pancreatic B-cell dysfunction [57], systemic inflammation [53], daytime fatigue [58], and appetite dysregulation [14,59]. OSA and obesity-related risk factors bidirectionally potentiate energy imbalance and excess adipose [58], further exacerbating the risk for glucose intolerance [55,60]. Both short sleep and OSA are hypothesized to elicit metabolic derangements including insulin resistance and glucose intolerance [61], independent of obesity [15].

Among the blood-based biomarkers, triglycerides and glucose were found to be significant correlates of AHI [62]. This result is in line with previous research connecting triglycerides with OSA [14,57,63]. Chou et al [13] identified the oxygen desaturation index, recorded as a component of polysomnography, as an independent risk factor for hypercholesterolemia and hypertriglyceridemia. Perry et al [63] demonstrated a dose-response relationship between chronic intermittent hypoxia and triglyceride levels using rodent experimentation. Although observational studies have identified cross-sectional relationships between short sleep and hypercholesterolemia [12,64], TST_7N_ was not related to the blood-based biomarkers within the current set of SLUMBRx-PONS participants. Sleep duration’s influence on hyperlipidemia has been posited to transpire through the activation of the sympathetic nervous system [64] and the concurrent catecholamine-induced lipolysis of free fatty acids [65]. As stress was not measured in the study sample, it could not be investigated as a mediator of TST_7N_ [66].

While short sleep is hypothesized to interact with fasting glucose, there was no correlation between glucose and TST_7N_ [61]. Analysis did identify an association between AHI and glucose, which aligns with research associating sleep-disordered breathing with glucose regulation [67]. The lack of correlation between glucose and TST_7N_ in our study may result from the small sample size. Previous research indicates that shorter sleep durations are associated with higher incidences of type 2 diabetes [68], likely through mechanisms involving pancreatic B-cell dysfunction [69], systemic inflammation [57], and metabolic derangements independent of obesity [7]. Given the smaller sample size of SLUMBRx-PONS, short sleep’s influence on metabolic indices may become more profound once the full sample is acquired.

### Limitations

Early findings from the SLUMBRx-PONS study should be interpreted considering several limitations. Although many of the findings from this study align with previous research, it is important to note the data were cross-sectional in nature, prohibiting the ability to infer temporal relationships between study variables. This is particularly relevant in the regression analyses. While the sleep indices were entered into the regression models as dependent variables, given the cross-sectional nature of the data, it is impossible to assert a temporal relationship between the sleep indices and the cardiometabolic disease risk factors. In addition, data were collected from a convenience sample of nonclinical participants. Given participants were not selected randomly, and were not randomized on study variables, caution is warranted when generalizing the results of this study. Although a sex-and-BMI-matching enrollment strategy was used, participants were not randomly selected from the population, limiting inference of the current findings on a broader scale. Furthermore, the smaller sample size limits the robustness of the current findings. In this study, short sleep was defined as less than 7 hours per night, a threshold supported by current literature [2]. However, further categorizing sleep duration into normal (7-8 hours), mild short (6 hours), moderate short (5 hours), and extreme short (3-4 hours) [47] could provide deeper insights into the risk implications of different sleep durations. Preliminary analyses did not show significant associations using these categories, likely due to the smaller sample size. Future analyses with a larger sample may yield more definitive results. Bootstrapping and composite measures derived from principal components were used to increase the robustness of the model CIs; nevertheless, this technique is only as valid as the initial sample collected.

There were also limitations attributed to methodological drawbacks common to clinical, epidemiological, and ambulatory data collection. Before beginning clinical assessment, participants were questioned about their dietary, exercise, and sleep behaviors during the previous 24 hours. Participants also provided their medical history and a list of all prescribed medications. Ambulatory data collection was susceptible to testing and social desirability biases [30]. It was assumed such effects would be minimal due to the 7-night timeframe in which sleep data were collected. There were also limitations inherent to sleep-measuring devices. HST and actigraphy allow for sleep duration and AHI data to be collected in a more naturalistic environment; however, they may lack the rigor offered by in-laboratory polysomnography [70,71]. Furthermore, the current findings are representative of an early cohort of healthy participants with normal baselines and the sample may not have yielded effect sizes strong enough to elicit positive results regarding the impact of OSA on risk factors for cardiovascular disease (eg, T90%). While actigraphy is generally an accepted standard for measurement of total sleep time, other home monitoring devices such as heart rate monitors, may offer more comprehensive data to increase measurement precision of sleep parameters [72]. Since one of the goals of the SLUMBRx-PONS study is to identify potential modalities and instruments for future community-based interventions, the limitations associated with these devices were deemed acceptable. Notwithstanding, FitBit devices are consumer-based products designed to assist with behavior modification. For the purposes of this study, participants were instructed to turn off the FitBit’s goal-setting features and requested not to track their health data on the FitBit phone app over the duration of the study.

### Conclusion

Early findings from the SLUMBRx-PONS study highlight significant associations between sleep indices and multiple cardiometabolic risk factors. The study’s limitations, including sample size and the inherent challenges of actigraphy and other ambulatory measures, are acknowledged. Ongoing efforts aim to increase participant numbers and improve the robustness of the current analyses. Future research will continue to explore the multi-factorial interactions between sleep, OSA, and cardiometabolic health, incorporating numerous measures such as T90% and measures of sleep fragmentation to enhance understanding.

## Abbreviations

AHI: apnea-hypopnea index
BCa: bias-corrected and accelerated
BP: blood pressure
DBP: diastolic blood pressure
HDL-C: high-density lipoprotein cholesterol (mg/dL)
HIPAA: Health Insurance Portability and Accountability Act
HST: home sleep test
LDL-C: low-density lipoprotein cholesterol (mg/dL)
OSA: obstructive sleep apnea
PAT: peripheral arterial tone
PCA: principal component analysis
PCAAHI×BMI: composite measure of apnea-hypopnea index and BMI derived from principal component analysis.
PCABP: composite measure of systolic blood pressure and diastolic blood pressure derived from principal component analysis
REI: Respiratory Event Index
SBP: systolic blood pressure
SLUMBRx-PONS: Short Sleep Undermines Cardiometabolic Health-Public Health Observational study
T90%: oxygen saturation below 90%
TST7N: Mean of 7 consecutive nights of total sleep time in minutes
*z*_GLU_: standardized mean of glucose
*z*_TRG_: standardized mean of triglycerides
*z*_TST7N_: standardized mean of 7 consecutive nights of total sleep time

## Data Availability

The data are not readily available because participants did not give consent to share data with other parties.
